# Cross Sectional Survey of Influenza Antibodies before and during the 2009 Pandemic in Shenzhen, China

**DOI:** 10.1371/journal.pone.0053847

**Published:** 2013-01-29

**Authors:** Chun-Li Wu, Juan Lu, Maggie Haitian Wang, Xing Lv, Ying Chen, Hsiang-fu Kung, Benny Zee, Xiao-wen Cheng, Ming-Liang He

**Affiliations:** 1 The Shenzhen Center for Disease Control and Prevention, Shenzhen, China; 2 Southern Medical University, Guangzhou, China; 3 Division of Biostatistics, School of Public Health and Primary Care, The Chinese University of Hong Kong, Hong Kong, China; 4 Stanley Ho Center for Emerging Infectious Diseases, School of Public Health and Primary Care, The Chinese University of Hong Kong, Hong Kong, China; 5 Li Ka Shing Institute of Health Sciences, The Chinese University of Hong Kong, Hong Kong, China; National Institutes of Health, United States of America

## Abstract

Much information is available for the 2009 H1N1 influenza immunity response, but little is known about the antibody change in seasonal influenza before and during the novel influenza A pandemic. In this study, we conducted a cross-sectional serological survey of 4 types of major seasonal influenza in March and September 2009 on a full range of age groups, to investigate seasonal influenza immunity response before and during the outbreak of the sH1N1 influenza in Shenzhen – the largest migration city in China. We found that the 0–5 age group had an increased antibody level for all types of seasonal influenza during the pandemic compared to the pre-outbreak level, in contrast with almost all other age groups, in which the antibody level decreased. Also, distinct from the antibodies of A/H3N2, B/Yamagata and B/Victoria that decreased significantly during the 2009 H1N1 pandemic, the antibody of A/H1N1 showed no statistical difference from the pre-outbreak level. The results suggest that the antibodies against the 2009 sH1N1 cross-reacted with seasonal H1N1. Moreover, the 0–5 age group was under attack by both seasonal and 2009 H1N1 influenza during the pandemic, hence vaccination merely against a new strain of flu might not be enough to protect the youngest group.

## Introduction

In 2009, a swine-origin H1N1 virus spread rapidly around the world. The initial outbreak occurred in April of that year in Mexico, and the World Health Organization (WHO) declared a global pandemic of the new type of influenza A in June 2009 [1]. By November 2009, 199 countries or regions had identified the virus in laboratory. Although the 2009 H1N1 virus (also referred as to swine flu, sH1N1) is antigenically different from previous seasonal influenza A (H1N1) [2], [3], there are increasing reports showing possible cross-reactivity of the antibodies to seasonal influenza antigens [4], [5], [6]. The natural immune response to the 2009 H1N1 has been extensively investigated [7], [8], and the status of the antibody against sH1N1 in risk populations before and after the pandemic has been repeatedly reported [9], [10]. However, few reports show the changes in seasonal influenza antibodies before and during the pandemic in risk populations, especially in Asia. In this study we conducted a cross-sectional serological survey of four major seasonal influenza types: A/H1N1, A/H3N2, B/Yamagata (B/Y) and B/Victoria (B/V) in March and September 2009, to investigate the seasonal influenza immunity response before and during the outbreak of the sH1N1 influenza. Cross-reactivity between antibodies of 2009 H1N1 and seasonal H1N1 is speculated. Also, comparisons show that the 0–5 age group antibody response is distinct from that of all other age groups in that its antibody response increased against all 4 types of seasonal influenza during the 2009 H1N1 pandemic from the pre-outbreak level. The 2009 H1N1 pandemic not only provided a major opportunity to elucidate the mechanisms of a new influenza strain transmission, outbreak and host response, but it also provided a new opportunity to study the mechanisms of the seasonal influenza switches. Such information will be very important for those who decide anti-influenza policy [11].

## Materials and Methods

### Geographical Background of the Study Area

Shenzhen, a Special Economic Zone opened up in the early 1980s for international trade, is the largest migration city in China. It is adjacent to Hong Kong and is a coastal city in Guangdong Province. Shenzhen has a population exceeding 14,000,000, of which more than 80% is non-residential (that is, the 80% comprises floating people who are working in Shenzhen with temporary resident permits). The mobility and high density of the population enable infectious diseases to be transmitted rapidly. As an international metropolis, about 0.2–0.3 million people travel to Shenzhen daily, either from Hong Kong or from other countries; thus, the control and prevention of infectious diseases is a demanding challenge for the city. The first incidence of 2009 H1N1 in Shenzhen was reported on 28 May 2009, and the peak of the pandemic occurred in September that year [12].

### Study Subjects I

#### Serum sampling

In this cross-sectional serological study, the study subjects were individuals with or without presence of influenza-like illness (ILI) who went to medical visit in hospitals in 7 districts of Shenzhen. They were recruited by stratified random sampling according to age groups: <5 years, 6–15 years, 16–25 years, 26–59 years, and above 60 years. In total 1,427 serum samples were collected from individuals aged from 0 to 85 during 2009, of which 535 were recruited in March (before the H1N1 pandemic) and 892 in September 2009 (during the H1N1 pandemic). On average, there were 48.6 males and 58.4 females in March, and 90.6 males and 87.8 females in September in each age group. The detailed information of each age group was listed in Table S1 and Table S2. The questionnaire included age, gender, history of respiratory tract infection, and history of vaccination and the presence or absence of ILI.

Based on the questionnaires, no participants recruited in this study had received vaccination against seasonal influenza during the period of 2006–2008. Informed consent from each study subject was collected in person or by the guardians. This study was approved by the Institutional Review Board and the Human Research Ethics Committee of the Shenzhen Center for Disease Control and Prevention (Shenzhen CDC). Written consent was obtained from the participants or the guardians of children.

#### Hemagglutination inhibition test

The human serum samples were treated with a receptor-destroying enzyme (Denka Seiken Co., Ltd, Tokyo, Japan) in a ratio of 4∶1 (volume: volume) at 37°C overnight to eliminate non-specific inhibitors of hemagglutination. Then the samples were tested for HA-specific antibodies by a standard hemagglutination-inhibition (HI) assay [13]. Two seasonal influenza A viruses (H1N1 and H3N2) and two seasonal influenza B viruses (B/Y and B/V) were used as antigens to measure the antibodies against each subtype of flu virus in the sera of cohorts. The tested seasonal strains were: A/Tianjin Jinnan/15/2009 (H1N1), A/Fujian Tongan/196/2009 (H3N2), B/Jiangxi Xiushui/32/2009 (Victoria), and B/Guangdong Xinxing/134/2009 (Yamagata). Serum-only controls for each human serum sample without added viral antigen were also assayed in parallel with the virus-specific assays. Only virus-specific assays with titer values greater than or equal to the corresponding serum-only control values were considered. An HI antibody titer of 1∶40 or more was considered seropositive. To calculate geometric mean titers (GMTs) for individual cohorts, titers below the lower limit (1∶10) were determined at the value of 1∶5 [14], [15]. The antibody titers used to calculate GMTs can be found in Supplementary Tables (Table S3, S4, S5, S6, S7, S8, S9, S10, S11, S12, S13, S14, S15, S16, S17, S18).

### Study Subjects II

In order to plot the overall trend of ILI incidences and influenza subtypes in 2009, we used the monthly data of ILI incidences and influenza subtypes tests provided by Shenzhen CDC. The test details are as follows:

#### Clinical nasopharyngeal swab specimens, virus culture and genotyping

Nasopharyngeal swab specimens were collected by the public health staff in the sentinel sites from ILI patients within three days of their illness having started but before any antiviral treatment of their symptoms had been initiated. The specimens were initially kept at 4°C. They were then transported twice a week to one of the virology laboratories maintained by the Shenzhen CDC and stored at −80°C for subsequent virus isolation and identification. The virus culture from the clinical samples was carried out either in MDCK cells for five to seven days or in embrocated chicken eggs for three days, as described previously [16]. The influenza-positive specimens were determined by a hemagglutination test (HA test) [17].

#### The genotypes and subtypes of the seasonal influenza

The influenza virus samples used in this study were collected as part of an ongoing national influenza surveillance program. The genotypes and subtypes were analyzed by an HA test using a WHO influenza diagnostic kit, and further confirmed by DNA sequencing, as described previously [18]. The monthly time series of the seasonal influenza was compiled by subtypes.

### Statistical Analysis

The common quantities used in serological analysis are the seropositivity rate and the geometric mean titer (GMT). GMT has the following expression:
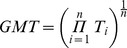



Where T_i_ is the HI titer, and n is the number of observations. However, when comparing two groups of HI titers using t-test, the GMT is likely to overestimate the difference, as t-test assumes a normal distribution but HI titers are on nonlinear fold-two scale. A log 2 transformation will put the HI titer data back to linear scale for comparison [19], [20], which takes expression as follows:
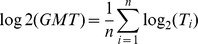



In the following analysis that compares antibody changes, the transformed data was used. To check the original GMT, the tabled value as an exponent of 2 can be used. A *p* value of <0.05 was considered statistically significant. The t-test was carried out in Microsoft Excel. Figures were plotted in R. Multivariate analysis was performed in IBM SPSS version 20.

## Results

### Comparison of Sera Antibody Titers between Influenza A and B

For Study Subjects I, in March, the antibody titers of seasonal influenza A were significantly higher than those of influenza B, whereas in September, there was no difference in antibody titers between the two types of influenza. In the 535 samples taken in March (229 male and 306 female), the log2 GMTs for A/H1N1, A/H3N2, B/Y and B/V were 3.572±1.313, 3.778±1.235, 4.279±1.591 and 3.905±1.725, respectively (Table 1). The titers of antibodies against influenza B viruses were significantly higher than those of influenza A by t-test (p-value = 0.0029). In September, from the data of 892 ILI patients comprising 454 males and 438 females, the GMTs in log2 scale for A/H1N1, A/H3N2, B/Y, and B/V were 3.452±1.272, 3.350±1.100, 3.536±1.272 and 3.582±1.144, respectively (Table 1). Although the antibody levels against influenza A viruses were slightly lower than those against influenza B viruses, there was no statistical difference. After making separate calculations for the male and the female groups, similar results were also observed (Table 2–3).

**Table 1:** General Comparison of Four Types of Seasonal Influenza Antibody Levels Before and During the 2009 H1N1 Influenza Pandemic (Mean titer level in log2 scale).

	A/H1N1	A/H3N2	B/Y	B/V
March	3.572±1.313	3.778±1.235	4.279±1.591	3.905±1.725
September	3.452±1.272	3.350±1.100	3.536±1.272	3.582±1.144
Difference	0.120	0.438	0.743	0.323
p-value	0.087	1.62×10^−11^	1.36×10^−21^	2.27×10^−5^
Bonferroni Adjusted P-value	0.348	6.48×10^−11^	5.44×10^−21^	9.08×10^−5^

Except for influenza type A/H1N1, the antibody levels of all 3 other seasonal influenzas significantly declined during the 2009 H1N1 pandemic compared to before the pandemic, using t-test.

**Table 2:** Comparison of Seasonal Influenza Antibody Change before and during the 2009 H1N1 Pandemic for Male (Mean titer level in log2 scale).

	A/H1N1	A/H3N2	B/Y	B/V
March	3.684	3.877	4.224	3.933
September	3.478	3.364	3.489	3.531
Difference	0.206	0.513	0.734	0.402
P-value	0.052	1.57×10^−7^	1.55×10^−11^	0.0003
Bonferroni Adjusted P-value	0.208	6.28×10^−7^	6.2×10^−11^	0.0012

Except for the seasonal A/H1N1 antibody, all other types of seasonal influenza antibodies significantly decreased in September in the male group.

**Table 3:** Comparison of Seasonal Influenza Antibody Change before and during the 2009 H1N1 Pandemic for female (Mean titer level in log2 scale).

	A/H1N1	A/H3N2	B/Y	B/V
March	3.489	3.704	4.322	3.884
September	3.425	3.336	3.585	3.635
Difference	0.064	0.369	0.737	0.249
P-value	0.499	9.99×10^−6^	3.32×10^−11^	0.018
Bonferroni Adjusted P-value	1	3.00×10^−5^	1.328×10^−10^	0.072

Except for the seasonal A/H1N1 antibody, all other types of seasonal influenza antibodies significantly decreased in September in the female group.

### Dissimilarity of Immunity Response of A/H1N1 and Other Seasonal Influenzas in the Presence of 2009 H1N1 Pandemic

In Table 1, except for seasonal H1N1, the antibodies of all other types of seasonal influenza (A/H3N2, B/Y and B/V) declined very significantly (*p*-value <10^−4^) during the 2009 H1N1 pandemic compared to the pre-outbreak level, whereas the antibodies of seasonal H1N1 only mildly decreased (*p*-value = 0.0873, Bonferroni adjusted *p*-value = 0.348). The dissimilarity of the antibody reaction of seasonal H1N1 and other seasonal influenzas is noteworthy, and we speculate that there might be cross-reactivity between the immunity responses of the two types of H1N1. Further investigation of the underlying mechanism was performed as follows.

### Analysis of the Trend of Influenza Incidences in Shenzhen, 2009

**Figure 1:**
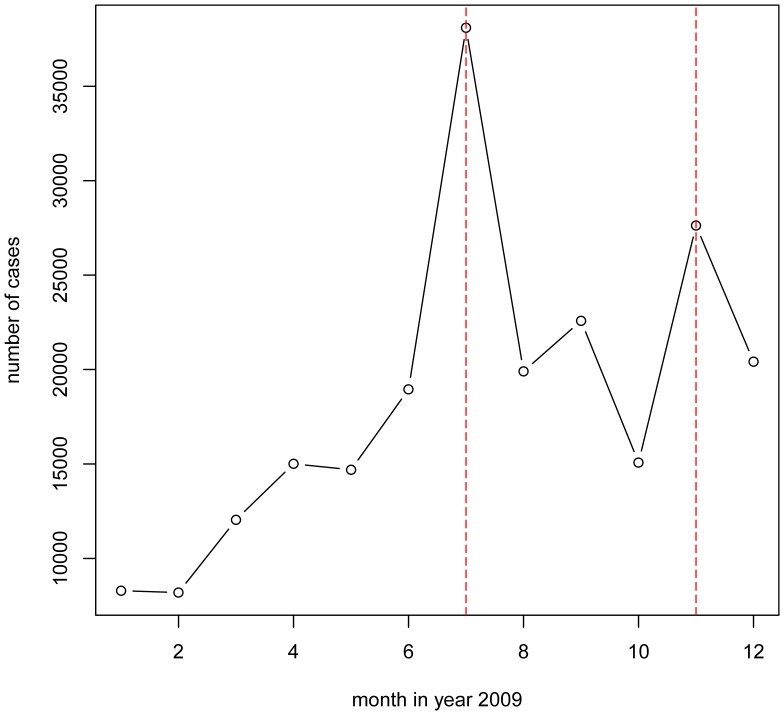
The total number of ILI cases in each month of 2009 in Shenzhen. In 2009, the peak of ILIs occurred in July 2009, sharply declined afterwards and formed a new wave in November. This may partially explain the significant drop in the three seasonal influenza antibody titer levels in September compared to March.

**Figure 2:**
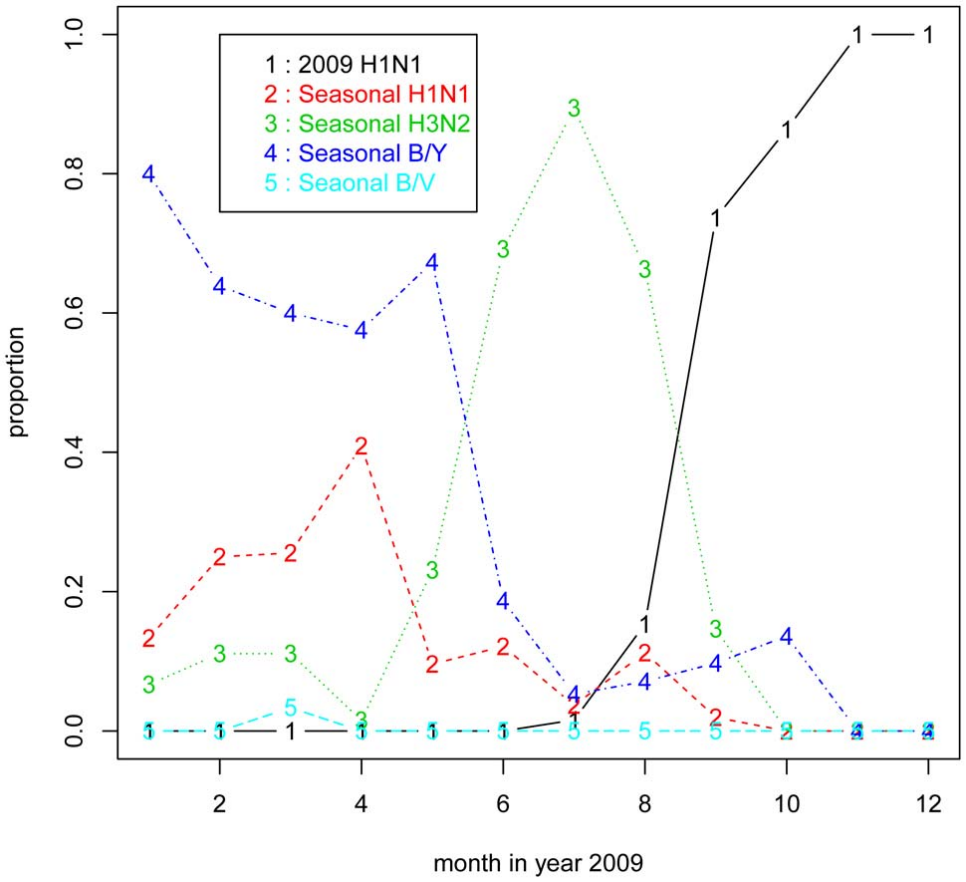
The proportion of each type of influenza in each month of 2009 in Shenzhen. The 2009 H1N1 influenza pandemic reached its peak in September, and dominated all ILIs in October, according to a survey of 5,125 subjects. Meanwhile, the seasonal H1N1 incidences decreased to a very low level in September, but its antibody titers stayed at a high level. The H3N2 peaked in July but rapidly decreased in August and September. This suggests that the seasonal H1N1 influenza antibody might have been present in sH1N1-infected cases, and could have been associated with the 2009 H1N1 antibody. The seasonal H1N1 antibody was therefore persistent during the pandemic peak of the 2009 H1N1 but after the peak of its own antigen.

We obtained the statistics of 220,883 influenza-like illness (ILI) cases in Shenzhen 2009 from study subjects II. The number of incidences is plotted on a month-by-month basis in Figure 1. The peak of ILIs occurred in July 2009, sharply declined afterwards, and formed a new wave in November. This could partially explain the significant drop of the three seasonal influenza antibody titer levels in September compared to March, but it could not explain the high level of A/H1N1 in September. From Study Subjects II, there are 5,125 incidences by influenza subtypes (Figure 2). It shows that the 2009 H1N1 pandemic peaked in September and dominated all ILIs in October. Moreover, the seasonal H1N1 incidences dropped off to a very low level in September. This fact, combined with the unusually high level of seasonal H1N1 antibody in September, implies that the seasonal H1N1 influenza antibody might have been present in swine H1N1-infected cases, and could have been associated with the 2009 H1N1 antibody. The seasonal H1N1 antibody was therefore persistent during the pandemic peak of the 2009 H1N1 but after the peak of its own antigen.

### Antibody Titer Change by Gender Groups

A comparison between gender groups was performed on the March and September data on Study Subjects I (In the remaining parts of the paper, all the analysis and results were based on Study Subjects I.) There was no significant difference between the seroprevalence in males and females in March or in September. All types of seasonal influenza antibodies significantly decreased in September in males and females except for the seasonal H1N1 antibody (Table 2–3), which is consistent with the previous results. However, the female group showed a more persistent antibody level of the seasonal H1N1 than the male group. In the case of females, the difference of mean titer level before and during the pandemic was 0.064; while for males, the difference was 0.206. A test on the differences gave *p*-value <10^−5^, and it supported the alternative hypothesis that male and female did not react the same during the pandemic. These results suggested that the seasonal H1N1 antibody was more sensitive in the male group, but more persistent in the female group during the 2009 H1N1 pandemic.

### Seasonal Influenza Antibody Prevalence in Age Groups

The highest seropositive rates were displayed in the 16–25 and the 26–59 age groups for almost all four types of seasonal influenza in March, but shifted to the 0–5 and the ≥60 age groups in September (Table 4 and 5). In particular, the 0–5 age group had a significantly elevated seropositive rate of seasonal H1N1 in September (28.4%), which was much higher than that of the other age groups (Table 5). It implies that the reactivity of seasonal H1N1 and 2009 H1N1 might be particularly strong in 0–5 year old children, or that pre-school age children were especially vulnerable to both types of H1N1 influenza during the 2009 H1N1 pandemic.

**Table 4:** Seropositive Rates in Each Age Group for Four Types of Seasonal Influenza in March.

Age group	A/H1N1	A/H3N2	B/Y	B/V
0–5	17.1%	9.8%	4.9%	13.8%
6–15	3.2%	6.5%	16.1%	4.8%
**16–25**	**25.3%**	**20.4%**	**59.9%**	25.3%
**26–59**	**24.8%**	**24.8%**	**48.1%**	**25.6%**
≥60	1.7%	15.3%	40.7%	**33.9%**
∑	18.1%	16.8%	37.2%	21.3%

Before the 2009 H1N1 influenza pandemic (March), the highest seasonal influenza prevalence age groups were 16–25 and 26–59 years old.

* **boldface** indicates the top two age groups with the highest seropositive rate.

**Table 5:** Seropositive Rates in Each Age Group for Four Types of Seasonal Influenza in September.

Age group	A/H1N1	A/H3N2	B/Y	B/V
**0–5**	28.4%	**15.4%**	14.9%	**17.9%**
6–15	3.6%	3.6%	5.4%	8.0%
16–25	12.9%	8.7%	**23.7%**	10.8%
26–59	11.2%	5.9%	12.8%	10.2%
**≥60**	**17.2%**	**18.5%**	**17.9%**	**18.5%**
∑	15.6%	10.7%	16.1%	13.2%

During the 2009 H1N1 pandemic (September), the highest seasonal influenza prevalence age groups was the age 0–5 group and the ≥60 age group.

* **boldface** indicates the top two age groups with the highest seropositive rate.

The seasonal influenza antibody level before and during the 2009 H1N1 pandemic is compared in Table 6, 7, 8, 9. To our surprise, the 0–5 age group and >60 age group had significantly *increased* seasonal A/H1N1 antibody levels during the pandemic, in contrast to all other age groups where the antibody level significantly declined. Moreover, the 0–5 age group had *increased* antibody for the other three types of seasonal influenza (A/H3N2, B/Yamagata and B/Victoria) during the pandemic compared to pre-pandemic levels, whereas all other age groups had a very significant drop in immunological response. This means that even during the epidemic of the new type of H1N1, the pre-school age children were very vulnerable to all types of seasonal influenza; thus, additional practices to protect this age group from both the new and conventional seasonal influenza should be carried out.

**Table 6:** Change of A/H1N1 Antibody Titer Level Between March and September by Age Group (mean titer value in log2 scale).

Age group/Group	0–5	6–15	16–25	26–59	≥60
March	3.533	3.306	3.779	3.663	3.169
September	3.874	2.983	3.347	3.349	3.534
Difference	**− 0.341**	0.323	0.432	0.314	**−0.365**
P-value	0.041	0.016	0.001	0.034	0.033
Bonferroni Adjusted P-value	0.205	0.08	0.005	0.17	0.165

Except for the 0–5 age group and a special case of >60 age in A/H1N1 influenza, all other age groups showed significantly decreased antibody levels of A/H1N1 during the 2009 H1N1 pandemic compared to before the pandemic, using t-test.

**Table 7:** Change of A/H3N2 Antibody Titer Level Between March and September by Age Group (mean titer value in log2 scale).

Age group/Group mean in log2 scale	0–5	6–15	16–25	26–59	≥60
March	3.590	3.709	3.878	3.772	3.966
September	3.625	2.884	3.260	3.204	3.640
Difference	**−0.035**	0.825	0.618	0.567	0.326
P-value	0.805	1.60×10^−7^	1.01×10^−7^	1.13×10^−5^	0.0618
Bonferroni Adjusted P-value	1	8×10^−7^	5.05×10^−7^	5.65×10^−5^	0.309

Except for the 0–5 age group, all other age groups showed significantly decreased antibody levels of A/H3N2 during the 2009 H1N1 pandemic compared to before the pandemic, using t-test.

**Table 8:** Change of B/Yamagata Antibody Titer Level Between March and September by Age Group (mean titer value in log2 scale).

Age group/Group mean in log2 scale	0–5	6–15	16–25	26–59	≥60
March	3.273	3.741	4.945	4.562	4.491
September	3.561	2.983	3.662	3.461	3.805
Difference	**−0.288**	0.759	1.283	1.101	0.686
P-value	0.0316	1.23×10^−6^	6.91×10^−15^	1.02×10^−11^	0.0009
Bonferroni Adjusted P-value	0.158	6.15×10^−6^	3.455×10^−14^	5.1×10^−11^	0.0045

Except for the 0–5 age group, all other age groups showed significantly decreased antibody levels of B/Y during the 2009 H1N1 pandemic compared to before the pandemic, using t-test.

**Table 9:** Change of B/Victoria Antibody Titer Level Between March and September by Age Group (mean titer value in log2 scale).

Age group/Group mean in log2 scale	0–5	6–15	16–25	26–59	≥60
March	3.769	3.451	4.020	3.872	4.390
September	3.874	3.072	3.463	3.472	3.898
Difference	**−0.105**	0.379	0.557	0.401	0.492
P-value	0.495	0.015	0.001	0.012	0.011
Bonferroni AdjustedP-value	1	0.075	0.005	0.06	0.055

Except for the 0–5 age group, all other age groups showed significantly decreased antibody levels of B/V during the 2009 H1N1 pandemic compared to before the pandemic, using t-test.

* **boldface** indicates an increased antibody level in September compared to that in March.

### Multivariate Analysis of the Relationship of sH1N1 Antibody Titer value, Gender, Age and Seasonal Influenza Antibody Titer Value

A multivariate analysis was performed using all Study Subjects I whose 2009 H1N1 HI antibody values were available. 2009 H1N1 antibody titers were used as response variable; gender, age and 4 seasonal influenza antibody titers were used as independent variables. All antibody titer values were in log transformed scale. Consistent with previous analysis, the result showed that seasonal A/H1N1 was significantly associated with 2009 H1N1 antibody with *p*-value <10^−5^ and further implied cross-reactivity between the two types of influenza antibodies. The other factors including gender, age, H3N2, B/Y and B/V were not found to be significant. Nevertheless, the *p*-values of age and H3N2 were 0.09. The complete output could be found in Table 10.

**Table 10:** Multivariate regression output: 2009 H1N1 antibody against gender, age, and seasonal influenza antibodies (log transformed scale).

Covariates	Beta	Std. Err	t	p-value
(Constant)	2.287	.126	18.096	.000
Gender	−.008	.069	−.117	.907
Age	.002	.002	1.511	.131
***H1N1***	**.** ***288***	**.** ***037***	***7.890***	**.** ***000***
H3N2	−.068	.040	−1.698	.090
B.Y	.045	.033	1.356	.175
B.V	−.021	.029	−.714	.475

## Discussion

Although the immunoresponse to the 2009 H1N1 has been extensively investigated, little is known about the antibody switches against the seasonal influenza subtypes during the sH1N1 pandemic. To fill the gap in our knowledge, this study investigated the serological response to the four types of seasonal influenza viruses, including the influenza A (A/H1N1, A/H3N2) and B (B/Yamagata, B/Victoria) viruses before and during the pandemic of the 2009 sH1N1 influenza in Shenzhen, the largest migration city in China.

There was no evidence that the seasonal H1N1 antibody changed in the pandemic from the pre-outbreak level, but the antibody of all three other types of seasonal influenza decreased significantly during the pandemic (Table 1). By further investigating the epidemics of the four types of seasonal influenza viruses, we showed that the ILIs, which were mainly composed of seasonal B/Y and seasonal H1N1 in March, decreased rapidly in August and September (Figure 1 and Figure 2). This partly explains why the antibodies in the patients against A/H3N2, B/Y and B/V decreased in September. It seems that the antibodies against 2009 H1N1 could cross-react with the seasonal influenza A/H1N1 because the antibodies against A/H1N1 were at similar levels both in March and September. This is not surprising because both the seasonal A/H1N1 and A/sH1N1 share much closer epitopes than the A/H3N2, B/Y and B/V subtypes. We also noted that the antibody titers of H3N2 markedly decreased in September compared to those in March, although the H3N2 went through a peak in July, and the infection rate in September was similar to that in March. However, the underlying mechanism was not clear.

There was no difference between the male and the female group in general; however, for seasonal A/H1N1, the antibody titer dropped much more in male than in female. It is generally reported that the number of incidences of the 2009 H1N1 infection was greater in males than in females; nevertheless, the severity of the infection was greater in the female cases [21]. A Canadian study reported that among the critically ill cases of 2009 H1N1, 74% of the deaths were female [22]. It may be suggested that females should be provided with greater protection against seasonal influenza virus infections. Of course, more data from other populations needs to be collected to confirm these phenomena.

When splitting the participants into age groups, we observed that the 15–25 and the 26–59 age groups had the highest seroprevalence of seasonal influenza before the pandemic, and during the pandemic the 0–5 and the ≥60 age groups had the highest seroprevalence. In particular, the 0–5 age group had increased antibody levels for all types of seasonal influenza during the pandemic, in contrast to almost all other age groups (except ≥60 age group in A/H1N1), in which the antibody level decreased. Cross-reactivity of the old and the new H1N1 antibody might be particularly strong in the 0–5 age group and the ≥60 age group. It also suggested that the youngest group had an especially high risk of being attacked both by the seasonal influenza and the 2009 H1N1 influenza during its pandemic. For all types of seasonal influenza, the 16–25 age group had the smallest decline in antibody levels during the pandemic compared to before the outbreak. The 0–5 age group data is especially valuable because in many studies this data is not available. The median infected cases’ age was around 40 [21], [23], [24], and the swine flu is understood to spread most virulently among young people. Consistent with our findings, in studies where the kindergarten children’s serological data are available, reports show that the 0–5 age group is still the primary risk population with the highest antibody response [25], [26], [27].

This study shows that during the 2009 H1N1 virus pandemic, all other seasonal influenza (A/H3N2, B/Y and B/Y) infections were suppressed. Based on the similarity of antigens between 2009 H1N1 and seasonal H1N1, it was also possible to posit that antibodies against the seasonal H1N1 could cross-react with sH1N1 and protected those exposed to the 2009 sH1N1. A multivariate analysis of 2009 H1N1 antibody titer with the 4 types of seasonal antibody titers resulted that the seasonal H1N1 influenza was the only significant (*p*-value <10^−5^) predictor of the pandemic antibody. The immunity generated in those who were newly exposed to the seasonal influenza viruses could possibly have played an important role in combating the 2009 sH1N1.

We have also shown a high antibody response to all seasonal influenza viruses in the 0–5 age group during the 2009 H1N1 pandemic; hence, vaccination against merely a new strain of flu may not be enough to protect the youngest age group during a new flu epidemic, but should be added to the existing seasonal influenza vaccination. Besides vaccination, extra protection such as early closure of day centers and primary schools should be carried out [28]. In future work, it would be informative to obtain the immunological response to the 2009 H1N1 before, during and after the outbreak, so that the pattern of its association to the seasonal H1N1 antibody could be studied, and prevention procedure, not only to the new influenza, but also to the existing seasonal ones, could be exercised.

## Supporting Information

Table S1 **Age and sex distribution of samples in March, 2009.**
(DOCX)

Table S2 **Age and sex distribution of samples in September, 2009.**
(DOCX)

Table S3 **Titre and age distribution of samples in March 2009 for serum antibodies against seasonal H1N1 by HI.**
(DOCX)

Table S4 **Titre and age distribution of samples in March 2009 for serum antibodies against seasonal H3N2 by HI.**
(DOCX)

Table S5 **Titre and age distribution of samples in March 2009 for serum antibodies against influenza B/Yamagata by HI.**
(DOCX)

Table S6 **Titre and age distribution of samples in March 2009 for serum antibodies against influenza B/Victoria by HI.**
(DOCX)

Table S7 **Titre and age distribution of samples in September 2009 for serum antibodies against seasonal H1N1 by HI.**
(DOCX)

Table S8 **Titre and age distribution of samples in September 2009 for serum antibodies against seasonal H3N2 by HI.**
(DOCX)

Table S9 **Titre and age distribution of samples in September 2009 for serum antibodies against influenza B/Yamagata by HI.**
(DOCX)

Table S10 **Titre and age distribution of samples in September 2009 for serum antibodies against influenza B/Victoria by HI.**
(DOCX)

Table S11 **2009 March H1N1 HI titer distribution.**
(DOCX)

Table S12 **2009 March H3N2 HI titer distribution.**
(DOCX)

Table S13 **2009 March B/Y HI titer distribution.**
(DOCX)

Table S14 **2009 March B/V HI titer distribution.**
(DOCX)

Table S15 **2009 September H1N1 HI titer distribution.**
(DOCX)

Table S16 **2009 September H3N2 HI titer distribution.**
(DOCX)

Table S17 **2009 September B/Y HI titer distribution.**
(DOCX)

Table S18 **2009 September B/V HI titer distribution.**
(DOCX)
