# Supplementary material for: Cross Sectional Survey of Influenza Antibodies before and during the 2009 Pandemic in Shenzhen, China
Source: PLoS One. 2013 Jan 29;8(1):e53847. doi: 10.1371/journal.pone.0053847 (PMC3558489; doi:10.1371/journal.pone.0053847)
Supplement: Table S1 — Age and sex distribution of samples in March, 2009. (DOCX) [file pone.0053847.s001.docx]

**Table S1** Age and sex distribution of samples in March, 2009

| Age groups (years) | Case number | Male | Female |
| --- | --- | --- | --- |
| 0-5 | 123 | 61 | 62 |
| 6-15 | 62 | 29 | 33 |
| 16-25 | 162 | 70 | 92 |
| 26-59 | 129 | 65 | 64 |
| ≥60 | 59 | 18 | 41 |
| ∑ | 535 | 243 | 292 |
