# Supplementary material for: Cross Sectional Survey of Influenza Antibodies before and during the 2009 Pandemic in Shenzhen, China
Source: PLoS One. 2013 Jan 29;8(1):e53847. doi: 10.1371/journal.pone.0053847 (PMC3558489; doi:10.1371/journal.pone.0053847)
Supplement: Table S2 — Age and sex distribution of samples in September, 2009. (DOCX) [file pone.0053847.s002.docx]

**Table S2** Age and sex distribution of samples in September, 2009

| Age groups (years) | Case number | Male | Female |
| --- | --- | --- | --- |
| 0-5 | 201 | 103 | 98 |
| 6-15 | 112 | 57 | 55 |
| 16-25 | 241 | 112 | 129 |
| 26-59 | 187 | 109 | 78 |
| ≥60 | 151 | 72 | 79 |
| ∑ | 892 | 453 | 439 |
