# Supplementary material for: Cross Sectional Survey of Influenza Antibodies before and during the 2009 Pandemic in Shenzhen, China
Source: PLoS One. 2013 Jan 29;8(1):e53847. doi: 10.1371/journal.pone.0053847 (PMC3558489; doi:10.1371/journal.pone.0053847)
Supplement: Table S3 — Titre and age distribution of samples in March 2009 for serum antibodies against seasonal H1N1 by HI. (DOCX) [file pone.0053847.s003.docx]

**Table S3** Titre and age distribution of **samples in March** 2009 for serum antibodies against **seasonal H1N1** by HI.

| Age group | GMT | Distribution of reciprocal antibody titres (# observations in each Titre category) | | | | | | |
| --- | --- | --- | --- | --- | --- | --- | --- | --- |
|  |  | <10 | 10 | 20 | 40 | 80 | 160 | 320 |
| 0-5 | 11.58 | 45 | 37 | 20 | 17 | 0 | 3 | 1 |
| 6-15 | 9.89 | 21 | 23 | 16 | 2 | 0 | 0 | 0 |
| 16-25 | 13.72 | 63 | 22 | 36 | 28 | 8 | 4 | 1 |
| 26-59 | 12.67 | 54 | 25 | 18 | 19 | 10 | 3 | 0 |
| ≥60 | 9.00 | 24 | 22 | 12 | 0 | 1 | 0 | 0 |
| ∑ | 11.90 | 207 | 129 | 102 | 66 | 19 | 10 | 2 |
