# Supplementary material for: Cross Sectional Survey of Influenza Antibodies before and during the 2009 Pandemic in Shenzhen, China
Source: PLoS One. 2013 Jan 29;8(1):e53847. doi: 10.1371/journal.pone.0053847 (PMC3558489; doi:10.1371/journal.pone.0053847)
Supplement: Table S4 — Titre and age distribution of samples in March 2009 for serum antibodies against seasonal H3N2 by HI. (DOCX) [file pone.0053847.s004.docx]

**Table S4** Titre and age distribution of **samples in March** 2009 for serum antibodies against **seasonal H3N2** by HI.

| Age group | GMTs | Distribution of reciprocal antibody titres(# observations in each Titre category) | | | | | | |
| --- | --- | --- | --- | --- | --- | --- | --- | --- |
|  |  | <10 | 10 | 20 | 40 | 80 | 160 | 320 |
| 0-5 | 12.04 | 34 | 49 | 28 | 6 | 1 | 1 | 4 |
| 6-15 | 13.08 | 11 | 25 | 22 | 2 | 0 | 1 | 1 |
| 16-25 | 14.70 | 37 | 45 | 47 | 23 | 7 | 2 | 1 |
| 26-59 | 13.66 | 43 | 26 | 28 | 24 | 7 | 1 | 0 |
| ≥60 | 15.63 | 7 | 21 | 22 | 5 | 3 | 1 | 0 |
| ∑ | 13.70 | 132 | 166 | 147 | 60 | 18 | 6 | 6 |
