# Supplementary material for: Cross Sectional Survey of Influenza Antibodies before and during the 2009 Pandemic in Shenzhen, China
Source: PLoS One. 2013 Jan 29;8(1):e53847. doi: 10.1371/journal.pone.0053847 (PMC3558489; doi:10.1371/journal.pone.0053847)
Supplement: Table S5 — Titre and age distribution of samples in March 2009 for serum antibodies against influenza B/Yamagata by HI. (DOCX) [file pone.0053847.s005.docx]

**Table S5** Titre and age distribution of **samples in March** 2009 for serum antibodies against **influenza B/Yamagata** by HI.

| Age group | GMT | Distribution of reciprocal antibody titres(# observations in each Titre category) | | | | | | |
| --- | --- | --- | --- | --- | --- | --- | --- | --- |
|  |  | <10 | 10 | 20 | 40 | 80 | 160 | 320 |
| 0-5 | 9.67 | 46 | 43 | 28 | 6 | 0 | 0 | 0 |
| 6-15 | 13.37 | 13 | 21 | 18 | 9 | 1 | 0 | 0 |
| 16-25 | 30.81 | 32 | 17 | 16 | 36 | 42 | 14 | 5 |
| 26-59 | 23.62 | 31 | 14 | 22 | 26 | 27 | 9 | 0 |
| ≥60 | 22.49 | 9 | 11 | 15 | 13 | 8 | 2 | 1 |
| ∑ | 19.41 | 131 | 106 | 99 | 90 | 78 | 25 | 6 |
