# Supplementary material for: Cross Sectional Survey of Influenza Antibodies before and during the 2009 Pandemic in Shenzhen, China
Source: PLoS One. 2013 Jan 29;8(1):e53847. doi: 10.1371/journal.pone.0053847 (PMC3558489; doi:10.1371/journal.pone.0053847)
Supplement: Table S6 — Titre and age distribution of samples in March 2009 for serum antibodies against influenza B/Victoria by HI. (DOCX) [file pone.0053847.s006.docx]

**Table S6** Titre and age distribution of **samples in March** 2009 for serum antibodies against **influenza B/Victoria** by HI.

| Age group | GMT | Distribution of reciprocal antibody titres(# observations in each Titre category) | | | | | | |
| --- | --- | --- | --- | --- | --- | --- | --- | --- |
|  |  | <10 | 10 | 20 | 40 | 80 | 160 | 320 |
| 0-5 | 13.63 | 27 | 54 | 25 | 7 | 3 | 1 | 6 |
| 6-15 | 10.94 | 11 | 35 | 13 | 3 | 0 | 0 | 0 |
| 16-25 | 16.22 | 69 | 30 | 22 | 13 | 3 | 0 | 25 |
| 26-59 | 14.64 | 51 | 29 | 16 | 10 | 13 | 3 | 7 |
| ≥60 | 20.96 | 10 | 14 | 15 | 10 | 4 | 4 | 2 |
| ∑ | 14.94 | 168 | 162 | 91 | 43 | 23 | 8 | 40 |
