# Supplementary material for: Cross Sectional Survey of Influenza Antibodies before and during the 2009 Pandemic in Shenzhen, China
Source: PLoS One. 2013 Jan 29;8(1):e53847. doi: 10.1371/journal.pone.0053847 (PMC3558489; doi:10.1371/journal.pone.0053847)
Supplement: Table S7 — Titre and age distribution of samples in September 2009 for serum antibodies against seasonal H1N1 by HI. (DOCX) [file pone.0053847.s007.docx]

**Table S7** Titre and age distribution of **samples in September** 2009 for serum antibodies against **seasonal H1N1** by HI.

| Age group | GMT | Distribution of reciprocal antibody titres(# observations in each Titre category) | | | | | | |
| --- | --- | --- | --- | --- | --- | --- | --- | --- |
|  |  | <10 | 10 | 20 | 40 | 80 | 160 | 320 |
| 0-5 | 14.66 | 69 | 46 | 29 | 31 | 19 | 3 | 4 |
| 6-15 | 7.90 | 59 | 36 | 13 | 4 | 0 | 0 | 0 |
| 16-25 | 10.19 | 113 | 54 | 43 | 21 | 7 | 2 | 1 |
| 26-59 | 17.55 | 81 | 50 | 35 | 14 | 5 | 2 | 0 |
| ≥60 | 11.85 | 57 | 36 | 32 | 21 | 5 | 0 | 0 |
| ∑ | 10.94 | 379 | 222 | 152 | 91 | 36 | 7 | 5 |
