# Supplementary material for: Cross Sectional Survey of Influenza Antibodies before and during the 2009 Pandemic in Shenzhen, China
Source: PLoS One. 2013 Jan 29;8(1):e53847. doi: 10.1371/journal.pone.0053847 (PMC3558489; doi:10.1371/journal.pone.0053847)
Supplement: Table S8 — Titre and age distribution of samples in September 2009 for serum antibodies against seasonal H3N2 by HI. (DOCX) [file pone.0053847.s008.docx]

**Table S8** Titre and age distribution of **samples in September** 2009 for serum antibodies against **seasonal H3N2** by HI.

| Age group | GMT | Distribution of reciprocal antibody titres(# observations in each Titre category) | | | | | | |
| --- | --- | --- | --- | --- | --- | --- | --- | --- |
|  |  | <10 | 10 | 20 | 40 | 80 | 160 | 320 |
| 0-5 | 12.34 | 64 | 57 | 49 | 20 | 8 | 3 | 0 |
| 6-15 | 7.38 | 73 | 19 | 16 | 4 | 0 | 0 | 0 |
| 16-25 | 9.59 | 103 | 77 | 40 | 16 | 4 | 1 | 0 |
| 26-59 | 9.22 | 81 | 60 | 35 | 9 | 2 | 0 | 0 |
| ≥60 | 11.58 | 47 | 41 | 35 | 24 | 4 | 0 | 0 |
| ∑ | 10.18 | 368 | 254 | 175 | 73 | 18 | 4 | 0 |
