# Supplementary material for: Cross Sectional Survey of Influenza Antibodies before and during the 2009 Pandemic in Shenzhen, China
Source: PLoS One. 2013 Jan 29;8(1):e53847. doi: 10.1371/journal.pone.0053847 (PMC3558489; doi:10.1371/journal.pone.0053847)
Supplement: Table S9 — Titre and age distribution of samples in September 2009 for serum antibodies against influenza B/Yamagata by HI. (DOCX) [file pone.0053847.s009.docx]

**Table S9** Titre and age distribution of **samples in September** 2009 for serum antibodies against **influenza B/Yamagata** by HI.

| Age group | GMT | Distribution of reciprocal antibody titres | | | | | | |
| --- | --- | --- | --- | --- | --- | --- | --- | --- |
|  |  | <10 | 10 | 20 | 40 | 80 | 160 | 320 |
| 0-5 | 11.80 | 75 | 52 | 44 | 17 | 9 | 2 | 2 |
| 6-15 | 7.90 | 63 | 31 | 12 | 5 | 1 | 0 | 0 |
| 16-25 | 9.59 | 90 | 64 | 30 | 38 | 13 | 3 | 3 |
| 26-59 | 11.01 | 64 | 65 | 34 | 18 | 5 | 0 | 1 |
| ≥60 | 13.98 | 36 | 47 | 41 | 21 | 1 | 2 | 3 |
| ∑ | 11.60 | 328 | 259 | 161 | 99 | 29 | 7 | 9 |
