# Supplementary material for: Cross Sectional Survey of Influenza Antibodies before and during the 2009 Pandemic in Shenzhen, China
Source: PLoS One. 2013 Jan 29;8(1):e53847. doi: 10.1371/journal.pone.0053847 (PMC3558489; doi:10.1371/journal.pone.0053847)
Supplement: Table S10 — Titre and age distribution of samples in September 2009 for serum antibodies against influenza B/Victoria by HI. (DOCX) [file pone.0053847.s010.docx]

**Table S10** Titre and age distribution of **samples in September** 2009 for serum antibodies against **influenza B/ Victoria** by HI.

| Age group | GMT | Distribution of reciprocal antibody titres | | | | | | |
| --- | --- | --- | --- | --- | --- | --- | --- | --- |
|  |  | <10 | 10 | 20 | 40 | 80 | 160 | 320 |
| 0-5 | 14.66 | 45 | 58 | 62 | 25 | 5 | 1 | 5 |
| 6-15 | 8.41 | 63 | 26 | 14 | 8 | 0 | 0 | 1 |
| 16-25 | 11.05 | 82 | 74 | 59 | 22 | 3 | 1 | 0 |
| 26-59 | 11.09 | 57 | 69 | 42 | 14 | 5 | 0 | 0 |
| ≥60 | 14.91 | 29 | 39 | 55 | 25 | 2 | 0 | 1 |
| ∑ | 11.98 | 276 | 266 | 232 | 94 | 15 | 2 | 7 |
