# Supplementary material for: Cross Sectional Survey of Influenza Antibodies before and during the 2009 Pandemic in Shenzhen, China
Source: PLoS One. 2013 Jan 29;8(1):e53847. doi: 10.1371/journal.pone.0053847 (PMC3558489; doi:10.1371/journal.pone.0053847)
Supplement: Table S11 — 2009 March H1N1 HI titer distribution. (DOCX) [file pone.0053847.s011.docx]

**Table S11 2009 March H1N1** HI titer distribution Male: 229 Female: 306

|  | GMT | Distribution of reciprocal antibody titres | | | | | | |
| --- | --- | --- | --- | --- | --- | --- | --- | --- |
|  |  | <10 | 10 | 20 | 40 | 80 | 160 | 320 |
| Male | 12.89 | 78 | 56 | 48 | 34 | 7 | 6 | 0 |
| Female | 11.22 | 129 | 73 | 54 | 32 | 12 | 4 | 2 |
