# Supplementary material for: Cross Sectional Survey of Influenza Antibodies before and during the 2009 Pandemic in Shenzhen, China
Source: PLoS One. 2013 Jan 29;8(1):e53847. doi: 10.1371/journal.pone.0053847 (PMC3558489; doi:10.1371/journal.pone.0053847)
Supplement: Table S12 — 2009 March H3N2 HI titer distribution. (DOCX) [file pone.0053847.s012.docx]

**Table S12 2009 March H3N2** HI titer distribution Male: 229 Female: 306

|  | GMT | Distribution of reciprocal antibody titres | | | | | | |
| --- | --- | --- | --- | --- | --- | --- | --- | --- |
|  |  | <10 | 10 | 20 | 40 | 80 | 160 | 320 |
| Male | 14.69 | 56 | 64 | 62 | 32 | 7 | 4 | 4 |
| Famale | 11.35 | 75 | 103 | 85 | 28 | 11 | 2 | 2 |
