# Supplementary material for: Cross Sectional Survey of Influenza Antibodies before and during the 2009 Pandemic in Shenzhen, China
Source: PLoS One. 2013 Jan 29;8(1):e53847. doi: 10.1371/journal.pone.0053847 (PMC3558489; doi:10.1371/journal.pone.0053847)
Supplement: Table S13 — 2009 March B/Y HI titer distribution. (DOCX) [file pone.0053847.s013.docx]

**Table S13 2009 March B/Y** HI titer distribution Male: 234 Female: 301

|  | GMT | Distribution of reciprocal antibody titres | | | | | | |
| --- | --- | --- | --- | --- | --- | --- | --- | --- |
|  |  | <10 | 10 | 20 | 40 | 80 | 160 | 320 |
| Male | 18.68 | 56 | 51 | 49 | 33 | 30 | 13 | 2 |
| Female | 20.00 | 75 | 55 | 50 | 57 | 48 | 12 | 4 |
