# Supplementary material for: Cross Sectional Survey of Influenza Antibodies before and during the 2009 Pandemic in Shenzhen, China
Source: PLoS One. 2013 Jan 29;8(1):e53847. doi: 10.1371/journal.pone.0053847 (PMC3558489; doi:10.1371/journal.pone.0053847)
Supplement: Table S14 — 2009 March B/V HI titer distribution. (DOCX) [file pone.0053847.s014.docx]

**Table S14 2009 March B/V** HI titer distribution Male: 229 Female: 306

|  | GMT | Distribution of reciprocal antibody titres | | | | | | |
| --- | --- | --- | --- | --- | --- | --- | --- | --- |
|  |  | <10 | 10 | 20 | 40 | 80 | 160 | 320 |
| Male | 15.28 | 70 | 71 | 35 | 23 | 10 | 1 | 19 |
| Female | 14.76 | 98 | 90 | 56 | 21 | 13 | 7 | 21 |
