# Supplementary material for: Cross Sectional Survey of Influenza Antibodies before and during the 2009 Pandemic in Shenzhen, China
Source: PLoS One. 2013 Jan 29;8(1):e53847. doi: 10.1371/journal.pone.0053847 (PMC3558489; doi:10.1371/journal.pone.0053847)
Supplement: Table S15 — 2009 September H1N1 HI titer distribution. (DOCX) [file pone.0053847.s015.docx]

**Table S15 2009 September H1N1** HI titer distribution Male: 454 Female: 438

|  | GMT | Distribution of reciprocal antibody titres | | | | | | |
| --- | --- | --- | --- | --- | --- | --- | --- | --- |
|  |  | <10 | 10 | 20 | 40 | 80 | 160 | 320 |
| Male | 11.14 | 191 | 114 | 76 | 43 | 22 | 6 | 2 |
| Female | 10.74 | 188 | 108 | 76 | 48 | 14 | 1 | 3 |
