# Supplementary material for: Cross Sectional Survey of Influenza Antibodies before and during the 2009 Pandemic in Shenzhen, China
Source: PLoS One. 2013 Jan 29;8(1):e53847. doi: 10.1371/journal.pone.0053847 (PMC3558489; doi:10.1371/journal.pone.0053847)
Supplement: Table S17 — 2009 September B/Y HI titer distribution. (DOCX) [file pone.0053847.s017.docx]

**Table S17 2009 September B/Y** HI titer distribution Male: 454 Female: 438

|  | GMT | Distribution of reciprocal antibody titres | | | | | | |
| --- | --- | --- | --- | --- | --- | --- | --- | --- |
|  |  | <10 | 10 | 20 | 40 | 80 | 160 | 320 |
| Male | 11.23 | 166 | 134 | 91 | 45 | 13 | 3 | 2 |
| Female | 12.00 | 162 | 125 | 70 | 54 | 16 | 4 | 7 |
