# Supplementary material for: Cross Sectional Survey of Influenza Antibodies before and during the 2009 Pandemic in Shenzhen, China
Source: PLoS One. 2013 Jan 29;8(1):e53847. doi: 10.1371/journal.pone.0053847 (PMC3558489; doi:10.1371/journal.pone.0053847)
Supplement: Table S18 — 2009 September B/V HI titer distribution. (DOCX) [file pone.0053847.s018.docx]

**Table S18 2009 September B/V** HI titer distribution Male: 454 Female: 438

|  | GMT | Distribution of reciprocal antibody titres | | | | | | |
| --- | --- | --- | --- | --- | --- | --- | --- | --- |
|  |  | <10 | 10 | 20 | 40 | 80 | 160 | 320 |
| Male | 11.56 | 146 | 147 | 103 | 43 | 11 | 1 | 3 |
| Female | 12.42 | 130 | 119 | 129 | 51 | 4 | 1 | 4 |
